# Examining the Relationship between Systemic Immune–Inflammation Index and Disease Severity in Juvenile Idiopathic Arthritis

**DOI:** 10.3390/cells13050442

**Published:** 2024-03-03

**Authors:** Delia-Maria Nicoară, Andrei-Ioan Munteanu, Alexandra-Cristina Scutca, Giorgiana-Flavia Brad, Iulius Jugănaru, Meda-Ada Bugi, Raluca Asproniu, Otilia Mărginean

**Affiliations:** 1 Department XI Pediatrics, Discipline I Pediatrics, ‘Victor Babeş’ University of Medicine and Pharmacy of Timisoara, 300041 Timisoara, Romania; nicoara.delia@umft.ro (D.-M.N.); scutca.alexandra@umft.ro (A.-C.S.); brad.giorgiana@umft.ro (G.-F.B.); juganaru.iulius@umft.ro (I.J.); asproniu.raluca@umft.ro (R.A.); marginean.otilia@umft.ro (O.M.); 2 Department of Pediatrics I, Children’s Emergency Hospital “Louis Turcanu”, 300011 Timisoara, Romania; bugi.ada@umft.ro; 3 Research Center for Disturbances of Growth and Development in Children BELIVE, ‘Victor Babeş’ University of Medicine and Pharmacy of Timisoara, 300041 Timisoara, Romania; 4 Ph.D. School Department, ‘Victor Babeş’ University of Medicine and Pharmacy of Timisoara, 300041 Timisoara, Romania

**Keywords:** Juvenile Idiopathic Arthritis, inflammation, cellular, systemic immune inflammation index, JADAS10

## Abstract

Juvenile Idiopathic Arthritis (JIA), the leading childhood rheumatic condition, has a chronic course in which persistent disease activity leads to long-term consequences. In the era of biologic therapy and tailored treatment, precise disease activity assessment and aggressive intervention for high disease activity are crucial for improved outcomes. As inflammation is a fundamental aspect of JIA, evaluating it reflects disease severity. Recently, there has been growing interest in investigating cellular immune inflammation indices such as the neutrophil-to-lymphocyte ratio (NLR) and systemic immune inflammation index (SII) as measures of disease severity. The aim of this retrospective study was to explore the potential of the SII in reflecting both inflammation and disease severity in children with JIA. The study comprised 74 JIA patients and 50 healthy controls. The results reveal a notable increase in median SII values corresponding to disease severity, exhibiting strong correlations with traditional inflammatory markers, including CRP and ESR (*ρ* = 0.714, *ρ* = 0.661), as well as the JADAS10 score (*ρ* = 0.690). Multiple regression analysis revealed the SII to be independently associated with JADAS10. Furthermore, the SII accurately distinguished patients with high disease activity from other severity groups (AUC = 0.827, sensitivity 81.5%, specificity 66%). These findings suggest that integrating the SII as an additional measure holds potential for assessing disease activity in JIA.

## 1. Introduction

Affecting up to 4 per 1000 children, JIA constitutes the most common rheumatic disease in children [1,2], being characterized by disordered immunity and chronic inflammation [3,4]. Generally acknowledged as a clinical syndrome comprising various disease subsets, it involves multiple inflammatory processes that converge into a common pathway [5,6]. Cases characterized by persistent active disease may lead to damage to articular cartilage and underlying bone [5,7,8]. Over the last decade, several advancements pertaining to the pathogenesis of JIA have led to the emergence of biological disease-modifying antirheumatic drugs, significantly improving disease outcomes [9,10,11]. Prompt diagnosis and therapeutic interventions are considered overarching principles in disease management [12,13]. Treatment decisions are guided by the ongoing and systematic assessment of disease activity [14]. In this regard, research has focused on identifying accurate disease activity measurements, with various clinical (various joint count types, pain measures, and global assessment scales) and laboratory (acute phase reactants, several cytokines) measures being proposed [15,16]. Aside from individual measures, composite disease activity measures, such as the Juvenile Arthritis Disease Activity Score (JADAS) and its variants, developed by Consolaro et al., have gained increased popularity [17,18,19].

Regarding laboratory measures, most of them have focused on quantifying protein responses to inflammation, such as C-reactive protein (CRP), the erythrocyte sedimentation ratio (ESR), and several antibodies [20,21,22,23]. However, there is a need for more nuanced assessments that go beyond traditional markers. In light of the advances in understanding the pathogenesis of JIA, which currently place more emphasis on innate immunity than in the past, it seems reasonable to consider evaluating indices that quantify the cellular response to inflammation as measures of disease activity [24,25]. In adult inflammatory arthritis, multiple studies have described the role of several cellular inflammation markers, such as the neutrophil-to-lymphocyte ratio (NLR), platelet-to-lymphocyte ratio, and systemic immune–inflammation index (SII), in reflecting both systemic inflammation and disease severity [25,26,27,28,29,30,31]. In children, there is a dearth of studies with a primary focus on the NLR [32]. However, research focusing on JIA’s pathogenesis has brought attention to the pivotal role of platelets in developing this disease [33,34]. The SII, a composite index that integrates the information of both NLR components and platelets, could, therefore, bring relevant information regarding the cellular response in JIA. Against this background, we sought to investigate the role of the SII as a potential inflammatory marker and its association with disease activity in children with JIA.

## 2. Materials and Methods

### 2.1. Study Design and Patient Selection

In this retrospective cross-sectional study, we enrolled children diagnosed with Juvenile Idiopathic Arthritis in the Rheumatology Department of the tertiary care Pediatric Emergency Hospital “Louis Turcanu” in Timisoara, Romania. We analyzed the medical charts of 93 consecutive patients who were admitted here during the period from January 2014 to October 2023. The inclusion criteria were (1) diagnosis of Juvenile Idiopathic Arthritis and (2) age under 18 years. Exclusion criteria were (1) systemic JIA, (2) arthritis due to any other illnesses, such as reactive arthritis, septic arthritis, vasculitis, acute rheumatic fever, malignancy, inflammatory bowel disease, and trauma; (3) active infections at the moment of admission or during the last two weeks prior to admission; and (4) patients with incomplete data. Upon review of the medical charts, we identified 74 patients who fulfilled the study criteria. All patients were diagnosed with Juvenile Idiopathic Arthritis according to the International League Associations for Rheumatology (ILAR) classification criteria [6]. Oligoarticular involvement was considered if fewer than five joints were affected, while polyarticular involvement was considered otherwise, based on the number of affected joints. In addition, 50 control subjects were included for comparison after reviewing their medical records. The exclusion criteria for control subjects comprised a diagnosis of inflammatory or autoimmune disease, acute or chronic infection, malignancy, and the use of medications known to affect complete blood count (CBC) parameters, such as corticosteroids.

This study received approval from the hospital’s Institutional Review Board and complied with the Declaration of Helsinki and its later amendments. Informed consent was waived due to the retrospective nature of the study.

### 2.2. Collection of Clinical Data and Assessment of Disease Activity

The following patient data were retrieved: age, gender, discharge diagnosis, ILAR category of JIA, disease duration, type of articular involvement, extra-articular disease manifestations, and current medication. Additionally, several disease activity parameters were recorded: active joint count, physician global rating of disease activity (measured on a 10 cm visual analog scale, where 0 means no activity and 10 signifies maximum activity), parent/patient global rating of wellbeing (measured on a 10 cm VAS, where 0 means very well and 10 very poor). Patients were categorized into three study groups according to their JADAS10 score [17]. The JADAS10 score was calculated as the arithmetic sum of the physician global rating of disease activity, parent/patient global rating of wellbeing, active joint count (with 10 as the maximum score in patients with 10 or more active joints), and the ESR, normalized to a 0–10 scale, using the formula (ESR-20)/10. The low-disease-activity (LDA) group comprised children with JADAS10 scores ranging from 1.1 to 2 for oligoarticular involvement and 1.1 to 3.8 for polyarticular involvement. The moderate-disease-activity (MDA) group consisted of children with JADAS10 scores ranging from 2.1 to 4.2 for oligoarticular involvement and 3.9 to 10.5 for polyarticular involvement. The high-disease-activity (HDA) group encompassed children with JADAS10 scores exceeding 4.2 for oligoarticular involvement and 10.5 for polyarticular involvement [35].

The blood samples collected upon hospital admission and analyzed for this study comprised a complete blood count (CBC) conducted using an automated hematology analyzer (Sysmex XN-550, Sysmex Corporation, Kobe, Japan) and CRP and ferritin performed using an automatic analyzer (Hitachi 747, Hitachi, Tokyo, Japan). Fibrinogen levels were determined using the Clauss method on an ACL Top Analyzer and ESR with the Westergren method. In addition, the following two hematological indices were computed based on the available CBC taken upon admission: the NLR (absolute neutrophil count/absolute lymphocyte count) and SII (absolute platelet count × NLR) [36,37].

### 2.3. Statistical Analysis

Statistical analyses were performed using the IBM Statistical Package for Social Sciences software (version 28, Armonk, NY, USA). The three study groups were characterized using descriptive statistics (percentage, median, range of quarters (IQR)). Visual (histograms, probability plots) and analytical methods (Shapiro–Wilk test) were employed to assess the normality of data distribution. Due to their abnormal distribution, numerical variables were expressed as medians (25th and 75th interquartile ranges (IQRs)) and compared using the Kruskal–Wallis test. Dunn’s test was conducted as a post hoc test to evaluate the statistical significance of distinctions among pairs of patient groups regarding SII values. Categorical variables were presented as numbers (percentages), and a Chi-squared test or Fisher’s exact test was performed, as appropriate, to compare these variables among research groups. The correlation between the two hematological indices, the NLR and SII, and several disease activity measures was evaluated using Spearman’s rank correlation coefficient (*ρ*). Linear regression was applied to identify associations between inflammation markers and JADAS10. For the univariate regression analysis, the concurrent medication variable was categorized into three groups: (1) no medication, (2) NSAIDs, and (3) immunosuppressants. While exploring predictor variables, we identified instances where certain combinations resulted in sparse data. Therefore, bootstrapping was performed to evaluate the robustness of the estimates and enhance the stability of our results. ROC curves were used to characterize the performance of several inflammatory markers in discriminating high disease activity. Youden’s index, calculated as sensitivity + specificity − 1, was used to estimate cutoff values for different biomarkers, while the area under the curve (AUC) in the ROC analysis was determined to compare the results. A *p*-value (two-tailed) <0.05 was deemed statistically significant.

## 3. Results

### 3.1. General Characteristics of the Study Population

Data from 74 children aged 1 to 18 years diagnosed with JIA were included in the present study, alongside 50 healthy controls matched for age and gender. The JIA patients were divided into three study groups based on disease activity status (35.1% with low disease activity, 28.4% with moderate disease activity, and 36.5% with high disease activity). Demographic data and disease characteristics are illustrated in Table 1. The median age of the entire study population was 13 [interquartile range (IQR): 9, 15.6] years, with a median disease duration of 1.2 (IQR: 0.6, 2.7) years. Gender distribution did not reveal significant variations across study groups (*p* = 0.136). Across the entire study population, the most common ILAR subtypes were enthesitis-related arthritis (ERA) (32.4.%) and oligoarticular JIA (29.7%); the majority of patients had oligoarticular involvement (60.8%), with no significant variations between disease activity groups. As expected, groups with more pronounced disease severity displayed a significant increase in all assessed disease activity parameters and biochemical inflammatory markers (Table 1).

### 3.2. Comparison of Hematological Parameters and Indices across Groups of Disease Activity

There was a significant gradual increase in the absolute count of white blood cells, neutrophils, and platelets with increased disease severity, as seen in Table 2. Conversely, hemoglobin levels were lower in the more severe study groups. The same trend of a gradual increase in disease severity was observed for both hematological indices, the NLR, and the SII (*p* < 0.001) across JIA groups. Compared to the control group, these differences were significant only regarding HDA and MDA patients. However, in comparing LDA and the control group, the only significant difference observed was a lower hemoglobin level among LDA patients. Nevertheless, there was a tendency towards a higher white WBC count, platelet count, and SII among LDA patients, although these differences did not reach statistical significance.

**Figure 1:**
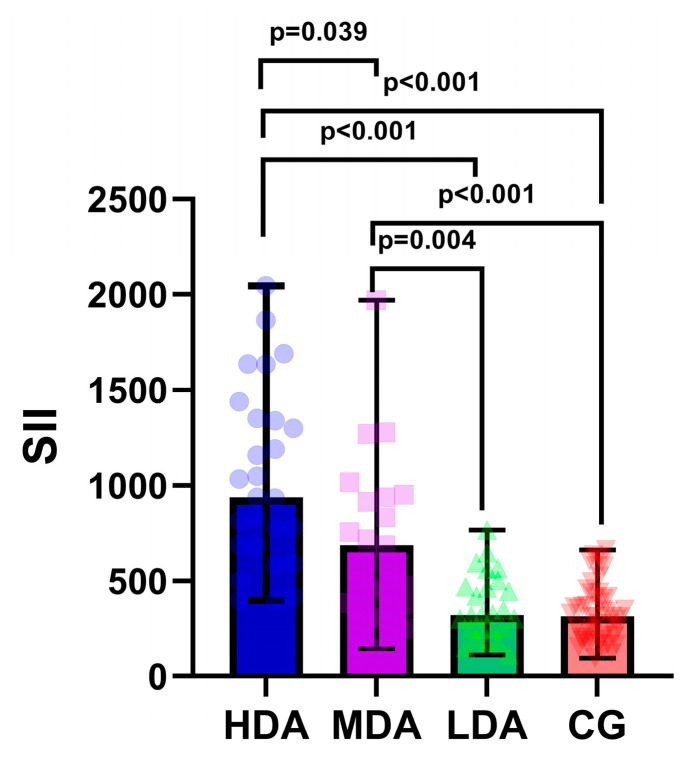
Boxplot diagram of systemic immune–inflammation index (SII) across study groups. LDA, low disease activity; MDA, moderate disease activity; HDA, high disease activity. The individual values are represented as semi-transparent circles, triangles, and rhombi.

As illustrated in Figure 1, there were significant differences in median SII values among all three study groups.

### 3.3. Correlation Analysis of Hematological Indices with Disease Activity Parameters

Spearman correlation analysis was performed to characterize the relationship between hematological indices, disease core set variables, and the JADAS10 score (Table 3). Significant positive correlations were observed between the NLR, SII, and all disease activity parameters, with a stronger correlation noted for the SII. The strongest correlation for both hematological indices was observed with CRP, while the weakest was observed with the active joint count. Regarding the median JADAS10 score, a strong correlation was observed exclusively with the SII (*ρ* = 0.697).

### 3.4. Relationship between SII and JADAS10

Furthermore, we employed linear regression to evaluate the association between the SII and JADAS10, as shown in Table 4. In the univariate analysis, we found associations between JADAS10 and the SII, CRP, and fibrinogen. However, following multiple linear regression, only CRP and the SII maintained significance as independent factors associated with JADAS10. Bootstrapping was employed to increase the stability of parameter estimates in the presence of a relatively limited number of observations.

**Figure 2:**
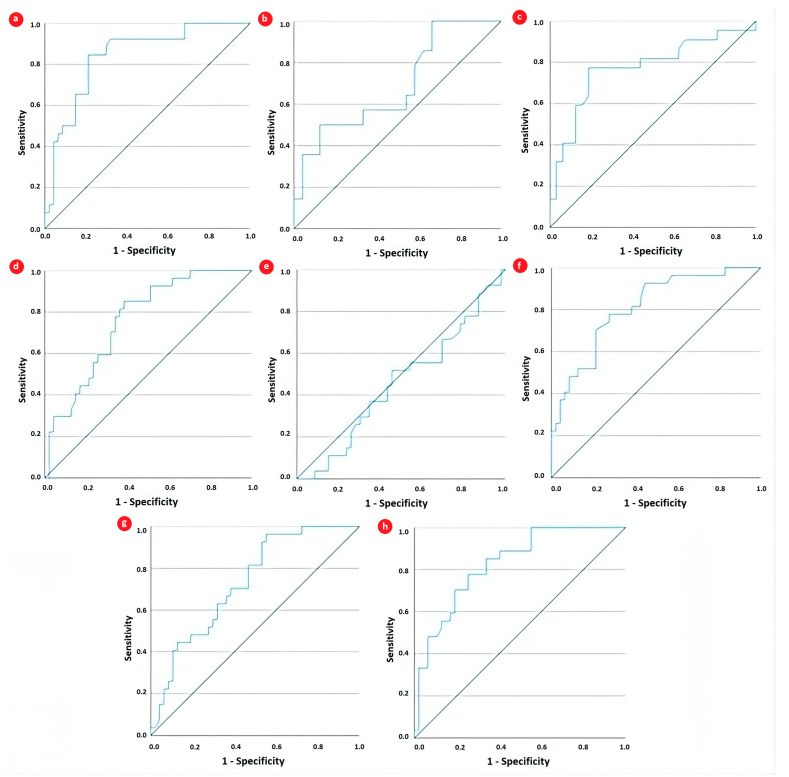
Receiver operating characteristic curve analysis for evaluating the performance of (**a**) CRP, (**b**) fibrinogen, (**c**) ferritin, (**d**) neutrophils, (**e**) lymphocytes, (**f**) platelets, (**g**) NLR, and (**h**) SII in discriminating high disease activity in JIA patients.

In addition, we assessed the diagnostic performance of the SII for identifying high disease activity by comparing it with various hematological parameters and the NLR. The AUC was calculated, and optimal cutoff values were determined using the Youden Index derived from the receiver operating characteristic (ROC) curve (Table 5). As illustrated in Table 5 and Figure 2, the most significant accuracy for high disease activity was displayed by CRP (AUC = 0.841, 81% sensitivity, and 79% specificity), followed closely by the SII (AUC = 0.827, 82% sensitivity, and 66% specificity). Platelet count also displayed borderline excellent discrimination ability (AUC = 0.809, 77% sensitivity, and 62% specificity), while neutrophils and the NLR presented acceptable discrimination ability (AUC = 0.729 and AUC = 0.761, respectively).

## 4. Discussion

In this retrospective study, we explored, for the first time, the clinical applicability of the SII in reflecting inflammatory burden and disease activity in real-world JIA patients. Our results show a significant positive correlation between the SII, a cellular inflammation marker, and disease activity markers, encompassing both clinical (JADAS10) and laboratory (CRP and ESR) measures. Furthermore, the SII exhibited excellent accuracy in distinguishing patients with high disease activity from other severity groups and demonstrated an independent, albeit modest, association with high disease activity in our study group.

Similar to other chronic diseases, JIA can manifest periods of disease activity and remission [38]. Ongoing advancements in JIA treatment have significantly improved the prognosis of this chronic condition, emphasizing the importance of early diagnosis and intervention as overarching principles in disease management [12,39]. There is a growing demand for more personalized treatment approaches, ensuring that children with unfavorable prognostic factors and those experiencing high disease activity receive early and aggressive interventions [40]. Consequently, the measurement of disease activity becomes a fundamental component in managing this condition [17]. Research efforts are focused on identifying the most effective measures of disease activity, a challenging task given the heterogeneity of JIA [41]. A proper evaluation of disease activity includes quantifying inflammatory responses [42]. Juvenile Idiopathic Arthritis, like most autoimmune rheumatic diseases, is accompanied by chronic inflammation [43]. This non-specific, multidimensional process is initiated, among other factors, by excessive production of inflammatory cytokines [44,45]. Most studies have focused on protein responses to pro-inflammatory cytokines, such as the conventional acute phase reactants CRP and ESR [20,21,22,23]. However, recent studies have raised concerns that ESR and CRP levels may not accurately reflect clinical disease activity in Juvenile Idiopathic Arthritis compared to their performance in adult inflammatory arthritis [46,47]. However, in addition to eliciting protein responses, inflammation also triggers cellular responses, causing changes in one or more cellular lineages within the hematopoietic system [48]. In contrast to earlier literature that emphasized dysregulated adaptive immunity, specifically the involvement of autoreactive Th1 and Th17 subsets, more recent studies highlight the significance of innate immunity in the immunopathology of JIA [24]. In this context, cellular responses to inflammation imply the release and migration of neutrophils and large platelets from the bone marrow to both circulating pools and sites of inflammation [49,50]. Due to limitations in accessing synovial inflammatory cells, particularly in children, some studies have shifted their focus to peripheral blood cells and observed changes in the biology of neutrophils and platelets in JIA patients [24,51,52,53]. In light of the role of blood cell interactions in inflammation and immune responses, several cellular immune inflammation markers have been shown to reflect a systemic inflammatory response [29,54]. These markers were initially studied in oncology and later extended to chronic inflammatory diseases, including rheumatic conditions [55,56,57,58]. Beyond the established role of the neutrophil-to-lymphocyte ratio, recent studies have investigated the applicability of the systemic immune–inflammation index in the context of inflammatory arthritis [26,31,59]. They found the SII was able to strongly predict disease activity, joint damage, and radiographic progression in rheumatoid arthritis [59]. Nevertheless, studies exploring its value in JIA are currently lacking.

To investigate the potential value of using the SII in children with JIA, we analyzed a study population stratified into low, moderate, and high disease activity according to the JADAS10 score. No statistically significant differences were found among the study groups concerning demographic characteristics (*p* = 0.569 for age, *p* = 0.230 for gender). In characterizing our patients, the most frequently observed ILAR subtypes were ERA (32.4%) and oligoarticular (29.7%), representing a notably higher rate of ERA compared to that found in most European epidemiological studies [60]. This discrepancy may stem from the tendency to refer more severe cases to our tertiary care center, while some of the milder oligoarticular cases are often managed on an outpatient basis in local healthcare centers.

As expected, cellular modifications reflecting the degree of systemic inflammation became progressively more evident with increasing disease severity. Peripheral leukocyte, neutrophil, and platelet counts gradually increased while hemoglobin levels decreased. This is in keeping with previous studies that noted neutrophilia and thrombocytosis as signs of active disease [61,62]. In our examination of the two hematological indices, both the NLR and SII showed a notable, gradual increase with the severity of the disease. The median NLR value of the entire study population was 1.63 (IQR: 1.19, 2.31), which is slightly smaller than the previous values reported in active JIA of 2.50 ± 1.89 [61] and 2.11 ± 1.19 [32]. The observed difference in NLR values may be influenced by the differing proportions of the JIA subtype and disease severity in the two studies and the difference in descriptive units. To the best of our knowledge, there are no reports of SII values in children with JIA. However, the SII value for our study population (592, IQR: 343, 942) was comparable to that reported by Satis et al. in adults with rheumatoid arthritis (667 ± 33) [54]. When comparing the CBC across study groups, statistically significant differences were observed between HAD, MDA, and controls. Additionally, a tendency towards a lower WBC count, platelet count, and SII was noted in the LDA group compared to the controls, although this difference did not reach statistical significance.

In order to determine whether the two hematological indices reflected the degree of systemic inflammation in our study population, we applied correlation analysis. We found both indices positively correlated with conventional inflammation markers, CRP, and ESR. However, the correlation was notably robust only for the SII, suggesting its potential as a reliable marker of inflammation. To further investigate the role of the SII in relation to disease activity, we performed a logistic regression analysis to ascertain its capacity to identify children with high disease activity. Timely identification of such patients allows for tailoring medications based on disease activity, leading to more optimal disease control. Multiple regression analysis revealed the SII to be independently associated with JADAS10. Furthermore, the SII demonstrated excellent predictive performance in identifying high disease activity status in ROC analysis. At a cutoff value of 586, it yielded a sensitivity of 81.5% and a specificity of 766%, with an AUC of 0.827, similar to CRP. As ESR was already incorporated into the JADAS10, which served as the classification criterion for disease activity status, we omitted it from regression and ROC analyses. In distinguishing children with high disease activity status, we also observed that the diagnostic AUC of the SII outperformed that of its individual component cell lineages. These findings suggest that the combined information provided by the SII may offer a more comprehensive assessment of inflammatory state and disease activity than its components in children with JIA. However, given the relatively small number of patients, the cutoff values cannot be generalized and should be regarded only as exploratory results.

It is important to consider potential limitations when interpreting the findings of the present study. First, due to the study’s retrospective nature, selection bias may be inherent. Second, the relatively small sample size of patients can be attributed to both the single-center design of the study and the lower frequency of the disease compared to adult arthritis. This may result in the limited extrapolation and robustness of the study results.

Furthermore, it restricted our ability to draw firm conclusions regarding the specific characteristics and outcomes associated with each subtype of JIA. Third, most patients were undergoing treatment, potentially influencing certain laboratory results; nonetheless, it is noteworthy that only 2 out of the 81 patients received oral corticosteroids in low doses at the time of the study. Therefore, prospective, multicenter studies would facilitate a more robust statistical assessment.

Our study also has several strengths. Firstly, as far as we know, this study is the first to assess the relationship between the SII and disease activity in patients with JIA. Secondly, patients reflect real-life settings, and the investigated hematologic index incurs no additional costs, as it is derived from the universally conducted complete blood count.

In conclusion, this study unveiled a gradual increase in the SII corresponding to disease severity in children with JIA. Moreover, the SII demonstrated an independent association with high disease activity status. In an era of ongoing efforts to explore chemokines and other biological markers, there is a tendency to somewhat downplay the significance of routine blood analyses, which are widely accessible. Given that not every healthcare center is equipped with advanced technology, we aimed to highlight the complementary value of routine blood work, such as the complete blood count. Considering that the SII reflects alterations in various inflammatory cell lineages implicated in the pathogenesis of JIA, our findings encourage us to consider the SII as an additional instrument in evaluating disease activity in children with JIA. Nevertheless, due to the relatively small sample size, our results should be regarded as exploratory rather than definitive, and additional multicentric studies are required to validate and reinforce these findings.

## Figures and Tables

**Table 1:** Demographic and clinical characteristics by disease activity status.

Variables	HDA (*n* = 27)	MDA (*n* = 21)	LDA (*n* = 26)	Control Group (*n* = 50)	*p*-Value
Demographic characteristics					
Age (years)	13.4 (9.11, 16.1)	11.3 (7.6, 14.6)	13 (8.6, 15.8)	13 (9, 14)	0.569 *^K^*
Disease duration (years)	1.1 (0.4, 3)	0.9 (0.4, 1.8) ^b^	1.5 (1, 3.9)		0.119 *^K^*
Females % (*n*)	55.6 (15)	71.4 (15)	42.3 (11)	53.8 (21)	0.230 ^χ2^
Joint involvement type % (*n*)					
Oligoarticular	55.6 (15)	57.1 (12)	69.2 (18)		0.547 ^χ2^
Polyarticular	44.4 (12)	42.9 (9)	30.8 (8)		0.547 ^χ2^
ILAR subgroups % (*n*)					
Oligoarticular	18.5 (5)	38.1 (8)	34.6 (9)		0.269 ^χ2^
Polyarticular, RF−	29.6 (8)	23.8 (5)	19.2 (5)		0.676 ^χ2^
Polyarticular, RF+	7.4 (2)	14.3 (3)	7.7 (2)		0.671 ^χ2^
ERA	40.7 (11)	23.8 (5)	30.8 (8)		0.450 ^χ2^
Psoriatic arthritis	18.5 (5)	0	7.7 (2)		0.411 ^χ2^
Extra-articular manifestations % (*n*)	25.9 (7)	14.3 (3)	23.1 (6)		0.275 ^χ2^
ANA positivity % (*n*)	7.4 (2)	19 (4)	7.7 (2)		0.356 ^χ2^
HLA B27 positivity % (*n*)	33.3 (9)	14.3 (3)	34.6 (9)		0.237 ^χ2^
Current medication % (*n*)	88.9 (24)	81 (17)	88.5 (23)		0.510 ^χ2^
NSAIDs	59.3 (16)	71.4 (15)	11.5 (3)		**<0.001** ^χ2^
Systemic steroids	7.4 (2)	0	0		0.167 ^χ2^
Conventional DMARD	22.2 (6)	9.5 (2)	73.1 (19)		**<0.001** ^χ2^
Biologic DMARD	7.4 (2)	0	34.6 (9)		**0.002** ^χ2^
Disease activity parameters (median, IQR)					
ESR (mm/h)	61 (34, 95) ^a,b^	22 (9.50, 30.5) ^b^	10 (5.75, 15.25)		**<0.001** *^K^*
CRP (mg/L)	15.5 (6.62, 52.8) ^a,b^	2.45 (0.61, 14.54) ^b^	0.91 (0.60, 1.99)		**<0.001** *^K^*
Active joint count	2 (1, 5)	1 (0, 1.5)	0 (0, 0)		**<0.001** *^K^*
Physician global	7 (6, 7)	4 (3.5, 5.5)	1 (1, 2)		**<0.001** *^K^*
Parental global	8 (7, 9)	5 (5, 6.5)	2 (1, 3)		**<0.001** *^K^*
JADAS-10	21.8 (18.4, 27) ^a,b^	11 (7.8, 16.2) ^b^	2 (1, 3.2)		**<0.001** *^K^*
Ferritin (ng/mL)	96 (30.7, 185.2)	46 (21, 70)	34 (15.5, 82.5)		0.140 *^K^*
Fibrinogen (mg/dL)	410 (348, 459) ^b^	356 (267, 425) ^b^	285 (255, 318)		**<0.001** *^K^*

*^K^* Kruskal–Wallis H-test and ^χ2^ Chi-square test. Data are expressed as median (interquartile range, IQR) or percentage (*n*, %). LDA, low disease activity; MDA, moderate disease activity; HDA, high disease activity; ILAR, International League of Associations for Rheumatology; RF, rheumatoid factor; ERA, enthesitis-related arthritis; ANA, antinuclear antibodies; HLA B27, human leukocyte antigen B27; NSAIDs, nonsteroidal anti-inflammatory drugs; DMARD, disease-modifying antirheumatic drug; ESR, erythrocyte sedimentation rate; CRP, C-reactive protein; VAS, visual analog scale; JADAS, Juvenile Arthritis Disease Activity Score. Statistically significant differences, meaning differences with a probability value of *p* < 0.05, are represented in bold. Compared with the MDA group, ^a^ *p* < 0.05. Compared with the HDA group, ^b^ *p* < 0.05.

**Table 2:** Hematological comparison by disease activity groups.

Variables	HDA (*n* = 27)	MDA (*n* = 21)	LDA (*n* = 26)	Control Group (*n* = 50)	*p*- Value
WBC ×10^3^ mm^3^	9.05 (7.36, 10.72) ^c,d^	8.92 (7.35, 10.84) ^c,d^	6.88 (5.33, 8.86)	6.65 (5.85, 8.40)	**<0.001**
Neutrophils ×10^3^ mm^3^	5.37 (4.44, 6.55) ^c,d^	5.59 (4.50, 6.12) ^c,d^	3.07 (2.25, 4.19)	3.11 (2.70, 3.87)	**<0.001**
Lymphocytes ×10^3^ mm^3^	2.68 (1.89, 3.31)	2.57 (1.93, 3.41)	2.66 (1.97, 3.52)	2.63 (2.17, 3.43)	0.729
Platelets ×10^9^ mm^3^	407 (376, 480) ^b,c,d^	342 (269, 402) ^d^	302 (261, 353)	272 (214, 312)	**<0.001**
Hemoglobin g/L	11.4 (10.4, 12.5) ^c,d^	12.5 (11.6, 12.8) ^d^	12.7 (11.8, 13.3) ^d^	13.5 (12.8, 14)	**<0.001**
NLR	2.13 (1.66, 3.05) ^c,d^	1.89 (1.27, 2.67) ^c,d^	1.12 (0.78, 1.55)	1.17 (0.91, 1.45)	**<0.001**
SII	938 (687, 1351) ^a,c,d^	685 (380, 946) ^c,d^	321 (234, 518)	315 (206, 421)	**<0.001**

Kruskal–Wallis H-test. Data are expressed as median (interquartile range, IQR). LDA, low disease activity; MDA, moderate disease activity; HDA, high disease activity; WBCs, white blood cells; NLR, neutrophil-to-lymphocyte ratio; SII, systemic immune–inflammation index. Statistically significant differences, meaning differences with a probability value of *p* < 0.05, are represented in bold. Compared with the MDA group, ^a^ *p* < 0.05. Compared with the HDA group, ^b^ *p* < 0.05. Compared with the LDA group, ^c^ *p* < 0.05. Compared with the control group, ^d^ *p* < 0.05.

**Table 3:** Correlation between hematological indices and disease activity indices in JIA.

Disease Activity Parameters	Hematological Indices			
	NLR		SII	
	*ρ*	*p*-Value	*ρ*	*p*-Value
ESR (mm/h)	0.512	**<0.001**	0.662	**<0.001**
CRP (mg/L)	0.676	**<0.001**	0.748	**<0.001**
Active joint count	0.352	**0.001**	0.426	**<0.001**
Physician global (10 cm VAS)	0.641	**<0.001**	0.714	**<0.001**
Parental global (10 cm VAS)	0.649	**<0.001**	0.742	**<0.001**
JADAS-10	0.594	**<0.001**	0.697	**<0.001**

ESR, erythrocyte sedimentation rate; CRP, C-reactive protein; VAS, visual analog scale; JADAS, Juvenile Arthritis Disease Activity Score. Statistically significant differences, meaning differences with a probability value of *p* < 0.05, are represented in bold.

**Table 4:** Regression analysis of factors related to JADAS10.

Variables	Univariate Analysis		Multivariate Analysis ^a^	
	B (95%CI)	*p*-Value	B (95%CI)	*p*-Value
Gender	1.154 (−3.346, 5.653)	0.611		
Age at onset	0.235 (−0.280, 0.750)	0.367		
ANA positivity	1.55 (−0.565, 8.762)	0.668		
Current medication	−3.194 (−6.441, 0.053)	0.054		
CRP	0.215 (0.161–0.269)	**<0.001**	0.094 (0.010, 0.178)	**0.029**
Fibrinogen	0.076 (0.056–0.097)	**<0.001**	0.024 (−0.010, 0.059)	0.160
SII	0.015 (0.011–0.018)	**<0.001**	0.008 (0.003, 0.014)	**0.004**

OR, odds ratio; CI, confidence interval; CRP, C-reactive protein; NLR, neutrophil-to-lymphocyte ratio; SII, systemic immune–inflammation index. ^a^ Bootstrap analysis, with results based on 1000 bootstrap samples. Statistically significant differences, meaning differences with a probability value of *p* < 0.05, are represented in bold.

**Table 5:** Comparison of hematological parameters and indices in discriminating high disease activity.

	AUC	SE	95%CI	Sensitivity	Specificity	Cutoff	*p*-Value
Neutrophils	0.729	0.058	0.616–0.843	0.704	0.532	4.48	0.001
Lymphocytes	0.457	0.070	0.321–0.594	0.444	0.543	2.80	0.545
Platelets	0.809	0.051	0.708–0.909	0.778	0.617	346	<0.001
NLR	0.761	0.055	0.653–0.869	0.741	0.660	1.73	<0.001
SII	0.827	0.047	0.734–0.920	0.815	0.660	586	<0.001
CRP	0.841	0.048	0.747–0.935	0.808	0.787	6.25	<0.001
Ferritin	0.689	0.091	0.512–0.866	0.643	0.417	33	0.055
Fibrinogen	0.773	0.070	0.636–0.910	0.727	0.812	360	0.001

NLR, neutrophil-to-lymphocyte ratio; SII, systemic immune–inflammation index; CRP, C-reactive protein; AUC, area under the curve; SE, standard error; 95%CI, 95% confidence interval. Statistically significant differences, meaning differences with a probability value of *p* < 0.05, are represented in bold.

## Data Availability

Data can be made available upon reasonable request due to ethical restrictions.
